# Extended Outbreak of Cryptosporidiosis in a Pediatric Hospital, China

**DOI:** 10.3201/eid1802.110666

**Published:** 2012-02

**Authors:** Yaoyu Feng, Lin Wang, Liping Duan, Luis A. Gomez-Puerta, Longxian Zhang, Xukun Zhao, Jingjing Hu, Nan Zhang, Lihua Xiao

**Affiliations:** East China University of Science and Technology, Shanghai, People’s Republic of China (Y. Feng, L. Wang, X. Zhao, J. Hu, N. Zhang);; Centers for Disease Control and Prevention, Atlanta, Georgia, USA (L. Wang, L. Duan, L.A. Gomez-Puerta, L. Xiao);; Henan Agricultural University, Zhengzhou, PRC (L. Zhang)

**Keywords:** *Cryptosporidium*, cryptosporidiosis, parasite, diarrhea, hospital-associated infection, outbreak, epidemiology, waterborne transmission, waterborne disease, genotyping, subtyping, China

## Abstract

Four *Cryptosporidium* spp. and 6 *C. hominis* subtypes were isolated from 102 of 6,284 patients in 3 pediatric hospitals in People’s Republic of China. A cryptosporidiosis outbreak was identified retrospectively. The outbreak lasted >1 year and affected 51.4% of patients in 1 hospital ward, where 2 *C. hominis* subtypes with different virulence were found.

Since the 1980s, ≈20 outbreaks of cryptosporidiosis have been reported in health care facilities (*1**–**9*). Thus far, to our knowledge, genotyping and subtyping tools have not been used in the investigation of this type of outbreak (*10*). We used subtyping in a molecular epidemiologic study of endemic cryptosporidiosis to retrospectively identify an extended outbreak among children in a hospital ward.

## The Study

During September 2007–October 2009, fecal specimens were collected from children in hospitals I (3,245 patients), II (489), and III (2,550), in Shanghai, People’s Republic of China. The children (1 month–19 years old, median 36 months) were hospitalized primarily for nongastrointestinal illnesses. For each patient, information was collected on age; sex; occurrence of diarrhea; and, later in the study, ward assignment in hospital I. The study was approved by the ethics committee of East China University of Science and Technology, Shanghai.

*Cryptosporidium* spp. were detected in the specimens and differentiated by PCR and restriction fragment length polymorphism analysis of the small subunit rRNA gene (*11*). *C. hominis* was subtyped by sequence analysis of the 60-kDa glycoprotein gene (*12*). Each specimen was analyzed at least 2× by PCR, with positive and negative controls in each run. Prevalence rates and 95% CIs were computed; the χ^2^ test was used to test differences. Odds ratios (ORs) and 95% CIs were calculated.

Among the 6,284 patients, 102 were positive for *Cryptosporidium* spp.: 90 from hospital I (2.8%, 95% CI 2.2–3.3), 3 from hospital II (0.6%, 95% CI 0–1.3), and 9 from hospital III (0.4%, 95% CI 0.1–0.6) (p<0.01). Ward assignment was available for 1,592 of 3,245 patients in hospital I. In most of the 12 wards, the infection rate was 0%–2.3%; in ward A, it was 51.4% (p<0.01) (Table).

**Table:** Distribution of *Cryptosporidium* spp. and subtypes among wards in a hospital in Shanghai, People’s Republic of China, September 2007–October 2009*

Ward	No. patient samples	% Positive (95% CI)	No. patients positive					
			*C. meleagridis*	*C. hominis*	IaA14R4	IdA19	IbA19G2	IdA14
A	74	51.4 (35.0–67.7)	0	38	19	14	0	0
B	348	0.6 (0–1.4)	1	1	0	1	0	0
C	283	1.8 (0.2–3.3)	2	3	0	1	0	1
D	216	2.3 (0.3–4.3)	1	4	2	1	1	0
E	266	1.1 (0–2.4)	0	3	0	1	0	0
F	56	1.8 (0–5.3)	0	1	0	0	0	0
Others	349	0	0	0	0	0	0	0
Unknown	1653	2.2 (1.5–2.9)	0	36	13	19	0	0
Total	3,245	28 (2.2–3.3)	4	86	34	37	1	1

*Among 86 specimens positive for *C. hominis*, 78 were gp60 positive and 73 were subtyped. In 2 other hospitals, II and III, among 6 *C. hominis*–specimens, 1 was IaA18R4 (hospital II), 1 was IgA14 (hospital III), 2 were IaA14R4 (hospital III), and 2 were gp60 negative (hospitals II and III); 2 patients each were positive for *C. meleagridis*, *C. canis*, and *C. felis*.

In hospital I, children <6 months old had a significantly higher positive rate (8.4%, 95% CI 5.6–11.2) than older children (1.9%, 95% CI 1.4–2.4) (p<0.01; data not shown). This was mainly because of a high infection rate among the age group in ward A (61.5%, 95% CI 36.9–86.2) versus those in other wards (40.0%, 95% CI 19.0–61.0). No age-associated difference in infection rates was found in other wards (p = 0.80; data not shown).

Cryptosporidiosis was more prevalent during February–July 2008 (p<0.01). Prevalence rates remained at ≈6% in the monthly distribution of the 2 main *C. hominis* subtypes in hospital I; however, when adequate numbers of patients were sampled, rates of *Cryptosporidium* infection in ward A remained >28% in most study months.

*C. hominis* was identified in 90.2% (92/102) of *Cryptosporidium*-positive patients in the 3 hospitals, of whom 86 were patients in hospital I. In hospital I, *C. hominis* was detected only in ward A; *C. meleagridis* was isolated from 4 patients in other wards (Table). In contrast, *C. canis* (1 case) and *C. hominis* (2 cases) were identified in hospital II patients, and *C. canis* (1 case), *C. hominis* (4 cases), *C. felis* (2 cases), and *C. meleagridis* (2 cases) were identified in hospital III patients (Table).

Six *C. hominis* subtypes were found at the 3 hospitals; 4 were in 73 specimens from hospital I (Table). Of those 73 specimens, 71 (97.3%) were subtype IaA14R4 or IdA19, and they were mostly found in ward A and unknown wards (Table). Other subtypes (IbA19G2 and IdA14) were not found in ward A (Table). With 1 exception, subtypes in hospital I were not found in other hospitals; subtype IaA14R4 was found in 2 patients in hospital III. Likewise, subtypes IaA18R4 (in 1 patient in hospital II) and IgA14 (in 1 patient in hospital III) were not found in hospital I.

In hospital I, 44 of 1,084 patients with diarrhea (4.1%, 95% CI 2.9–5.3) and 46 of 2,161 without diarrhea (2.1%, 95% CI 1.5–2.7) were positive for *Cryptosporidium* spp. (p = 0.002, OR 1.95, 95% CI 1.28–2.96). *C. hominis* subtype IaA14R4 (21 diarrheic and 13 nondiarrheic cases) was significantly associated with diarrhea (p = 0.0004, OR 3.29, 95% CI 1.64–6.59), but subtype IdA19 (11 diarrheic and 26 nondiarrheic cases) was not (OR 0.86, 95% CI 0.42–1.75, p = 0.68).

## Conclusions

Our data indicate that a cryptosporidiosis outbreak occurred among children in ward A of hospital I. This conclusion was supported by the following findings: the rate of *Cryptosporidium*-positive cases in ward A (51.4%) was significantly higher than the overall rates in hospitals I (2.8%), II (0.6%), and III (0.4%); less *Cryptosporidium* diversity was found in ward A (only *C. hominis*) than in other wards/hospitals (4 *Cryptosporidium* spp.); only *C. hominis* subtypes IaA14R4 and IdA19 were present among 38 ward A patients (vs. 6 subtypes in 12 patients in other wards/hospitals); and a high rate (61.5%) of *Cryptosporidium*-positive cases occurred in ward A among children <6 months old, an age that usually has a low prevalence of cryptosporidiosis (*13*).

The source of the cryptosporidiosis outbreak is unknown. Most of the 12 wards in hospital I were located in the main building; ward A, the smallest ward, was in an adjacent building and was for children from a welfare institute. Hired caregivers cared for children in ward A; family members were the primary caregivers for patients in other wards. Thus, poor diaper-changing and hand-washing practices by caregivers could be responsible for the persistence of *C. hominis* infections in ward A. However, the facts that most of the patients were examined for *Cryptosporidium* infection only once and that many of the specimens were not submitted immediately after patients were hospitalized prevented us from concluding with certainty whether the infections were acquired in the hospital or in the welfare institute. The likelihood for widespread foodborne and waterborne transmission of cryptosporidiosis in hospital I was small because children in ward A and other wards shared the same source for food and drinking water. The likelihood of direct transmission of cryptosporidiosis among ward A patients was also small because 80% of patients were <1 year old and mostly stayed in cribs and beds.

This cryptosporidiosis outbreak has several key features. First, it was lengthy, lasting >14 months (November 2007–December 2008); only limited sampling was done before November 2007; and *Cryptosporidium* spp. were still present in December 2008. The longest previous outbreak was 4 months (*14*). Second, the number (>38) of involved patients was high. Judged by the low occurrence of the 2 subtypes in other wards, most of the 32 IaA14R4- and IdA19-positive patients with missing ward information were probably also from ward A. Thus, >60 children might have been part of the outbreak. Third, this outbreak was caused concurrently by 2 *C. hominis* subtypes, of which IaA14R4, but not IdA19, was significantly associated with diarrhea. The observed difference in virulence is consistent with data from a community study in Peru (*15*), in which subtype family Ia, of which IaA14R4 is a member, was more virulent than Id, of which IdA19 is a member.

We retrospectively identified the outbreak by subtyping; the delay in detection prevented us from doing a thorough investigation, and continued sampling in the hospital and welfare institute and detailed epidemiologic and environmental investigation became impossible after we reported the outbreak to hospital I. Despite not knowing the source of infections, hospital I took measures to reduce hospital-acquired infections, including better training of caregivers and moving ward A to a new location. Thus, study data highlight the power of molecular epidemiologic tools in the surveillance and control of cryptosporidiosis and the need for prompt identification and investigation of outbreaks in health care facilities.
